# The association between serum albumin and alkaline phosphatase in cancer patients

**DOI:** 10.1097/MD.0000000000037526

**Published:** 2024-03-29

**Authors:** Yiqian Jiang, Yong Cai, Yingying Ding, Xiangyang Kong, Zhaoyang Li

**Affiliations:** a Department of Radiotherapy, Xiaoshan Affiliated Hospital of Wenzhou Medical University, Hangzhou, Zhejiang, China; b Department of pediatrics, Xiaoshan Affiliated Hospital of Wenzhou Medical University, Hangzhou, Zhejiang, China; c Department of Respiratory, Xiaoshan Affiliated Hospital of Wenzhou Medical University, Hagnzhou, Zhejiang, China; d Department of Oncology, Affiliated Hospital of Hangzhou Normal University, Hangzhou, Zhejiang, China.

**Keywords:** albumin, alkaline phosphatase, bone metastases, NHANES

## Abstract

The role of serum albumin (ALB) has been extensively studied in patients with cancer; however, research on its effect on bone metastasis in these patients remains limited. This study aimed to investigate the relationship between serum ALB and alkaline phosphatase (ALP) levels in patients with tumors. Using data from the National Health and Nutrition Examination Survey 2011 to 2018, we assessed the correlation between serum ALB and ALP levels using a weighted multivariate linear regression model, whereas a weighted generalized additive model and smooth curve fitting were used to address potential nonlinearities. A total of 1876 patients with cancer were included in our study. In the subgroup analysis stratified by sex, race/ethnicity, and liver disease, the negative correlation of ALB with ALP remained for most groups, except in blacks (β = −1.755, 95%CI: [−3.848, 0.338], *P* = .103) and patients with gout (β = −0.676, 95%CI: [−2.061, 0.709], *P* = .340). In black people and patients with gout, the relationship between ALB and ALP showed an inverted U-shaped curve, with an inflection point at approximately 42 g/dL. Our study showed an inverse correlation between ALB and ALP levels in most patients with tumors, but not in black patients and those with gout. The measurement of ALB levels can serve as a screening tool for bone metastases while guiding therapeutic intervention strategies.

## 1. Introduction

According to the World Health Organization, cancer is a serious health problem worldwide. Its incidence continues to increase owing to various risks such as genetic mutations, environmental pollution, and aging populations.^[1,2]^ Therefore, cancer remains one of the leading causes of death worldwide and is a major obstacle to increasing global life expectancy in the 21st century.^[3,4]^ Bone metastasis can occur in many types of solid tumors. Bone-related events caused by bone metastases, such as bone pain, pathological fractures, spinal cord compression, and hypercalcemia, seriously affect the quality of life of patients with cancer, weaken the body response to tumor resistance, and lead to decreased survival.^[5]^ Serum alkaline phosphatase (ALP) is mainly secreted by osteoblasts and can also be produced by the kidneys, gastrointestinal tract, and other organs. Currently, ALP has been used as an important indicator of bone metastases in patients with cancer.^[6]^ Bone metastases are closely associated with bone metabolic disorders. Consequently, ALP is highly expressed in the serum of patients with bone metastases; the higher the degree of bone metastasis, the higher the level of ALP.^[7,8]^ The predictive sensitivity, specificity, and accuracy of ALP in cancer bone metastases are over 70%; therefore, it has important value in the prediction of tumor bone metastases and is an independent prognostic indicator in patients with bone metastasis.^[9,10]^

Serum albumin (ALB) is a single polypeptide chain synthesized by hepatocytes that consists of 585 amino acid residues and is the main component of serum proteins. ALB has various functions, such as maintaining plasma colloid osmotic pressure, transporting nutrients, and anti-oxidative effects.^[11]^ Studies have found that malnutrition can promote the occurrence and development of tumors and has an impact on the treatment and survival of patients.^[12,13]^ Serum ALB levels are an important indicator of body nutrition, immune status, and surgical risk. Studies have confirmed that a decrease in serum ALB levels is closely related to poor prognosis in patients with colon cancer, lung cancer, and other malignant tumors.^[14,15]^ Although the ALB/ALP ratio has been extensively studied in malignant tumors,^[16,17]^ studies on the association between ALB and ALP are limited, especially in patients with malignant tumors. Therefore, we investigated the relationship between ALB and ALP levels in a representative sample of patients with malignancies using the 2011 to 2018 National Health and Nutrition Examination Survey (NHANES) database.

## 2. Materials and methods

### 2.1. Study population

The NHANES is a nationally representative survey of the United States population that uses a complex, multistage, probability sampling design to provide extensive information on nutrition and health.^[18,19]^ Our study analyzed data from the NHANES over an 8 year period (2011–2018). A total of 2172 patients with cancer between the ages of 20 and 80 years that were included in the NHANES 2011 to 2018 database were enrolled. After excluding 294 participants without ALB data and 2 without serum ALP data, 1876 subjects remained in the final analysis.

The data used in this study were obtained from a public database (https://www.cdc.gov/nchs/nhanes/index.htm). The National Center for Health Statistics Ethics Review Board approved all NHANES protocols, and the participants or their proxies provided informed consent prior to participation.^[20]^

### 2.2. Study variables

The exposure variable was the ALB level. ALB concentration was measured using the DcX800 method, which is a bichromatic digital endpoint method. The outcome variable was bone ALP levels. The ALP measurement method utilizes a simple colorimetric reaction, wherein ALP acts on a substrate (p-nitrophenol phosphate or PNPP) in the presence of magnesium and zinc activators to form a colored product (p-nitrophenol), whose appearance is measured at 450 nm. The following covariates were included: age, sex, race/ethnicity, alanine aminotransferase, blood urea nitrogen, creatine phosphokinase, cholesterol, creatinine, gamma-glutamyl transferase, glucose, phosphorus, total protein, uric acid, globulin, triglycerides, body mass index, aspartate aminotransferase, iron, lactate dehydrogenase, arthritis, gout, and liver disease. Details of the serum ALB and bone ALP measurement processes and other covariate acquisition processes are available at www.cdc.gov/nchs/nhanes/.

### 2.3. Statistical analyses

All analyses were performed using R software (version 3.4.3) and EmpowerStats software (http://www.empowerstats.com), with statistical significance set at *P* < .05. We used the weighted *χ*^2^ test for categorical variables and the weighted linear regression model to calculate the association between ALB and bone ALP using 3 models: an unadjusted model; minimally adjusted model, controlling for sex race and age; and a fully tuned model. A subgroup analysis was performed using stratified multivariate linear regression models. We further explored the potential nonlinear relationship between ALB and bone ALP levels by using a generalized additive model and smooth curve fitting.

## 3. Results

The baseline characteristics of the 1876 patients with cancer included in our study, categorized by quartiles of serum ALB, are shown in Table 1. These characteristics differed significantly between the ALB quartiles, except for creatine phosphokinase, uric acid, and gout. Compared with other subgroups, participants in the highest ALB quartile were more likely to be male and non-Hispanic white, with higher values of alanine aminotransferase, cholesterol, phosphorus, total protein, and iron, and lower values of bone ALP, blood urea nitrogen, glucose, globulin, body mass index, and age.

**Table 1:** Characteristics of the study population based on serum albumin quartiles.

Albumin (g/dL)	Q1 (20–38)	Q2 (39–41)	Q3 (42–43)	Q4 (44–52)	*P* value
N	348	581	438	509	
Age (yr)	67.26 ± 13.39	66.83 ± 13.28	65.37 ± 13.04	63.58 ± 14.89	<.001
ALT (IU/L)	19.44 ± 12.67	21.23 ± 13.11	23.56 ± 14.10	24.93 ± 18.55	<.001
ALP (IU/L)	88.52 ± 51.75	74.47 ± 24.33	70.92 ± 23.70	68.37 ± 22.89	<.001
Blood urea nitrogen (mg/dL)	6.73 ± 3.77	6.11 ± 2.57	5.82 ± 2.36	5.70 ± 2.42	<.001
CPK (IU/L)	126.49 ± 269.27	118.92 ± 106.67	124.97 ± 99.23	144.77 ± 210.57	.102
Cholesterol (mmol/L)	4.51 ± 1.07	4.90 ± 1.11	5.03 ± 1.26	5.14 ± 1.14	<.001
Creatinine (µmol/L)	108.88 ± 106.18	86.27 ± 33.45	82.41 ± 26.16	84.95 ± 28.44	<.001
GGT (IU/L)	33.36 ± 48.05	28.39 ± 30.91	26.45 ± 25.10	27.97 ± 27.83	.025
Glucose (mmol/L)	6.51 ± 3.01	6.26 ± 2.60	5.91 ± 1.98	5.58 ± 1.23	<.001
Phosphorus (mmol/L)	1.17 ± 0.23	1.19 ± 0.18	1.20 ± 0.18	1.22 ± 0.18	.009
Total protein (g/L)	67.76 ± 5.58	69.22 ± 4.73	70.01 ± 3.99	71.98 ± 4.19	<.001
Uric acid (mg/dL)	5.69 ± 1.67	5.61 ± 1.48	5.55 ± 1.41	5.51 ± 1.42	.370
Globulin (g/L)	31.49 ± 5.98	29.06 ± 4.78	27.50 ± 4.01	26.43 ± 4.04	<.001
Triglycerides (mmol/L)	1.61 ± 0.97	1.77 ± 1.25	1.89 ± 2.03	1.78 ± 1.15	.066
BMI (kg/m^2^)	31.21 ± 8.26	30.36 ± 6.88	28.90 ± 5.61	27.22 ± 5.30	<.001
AST (IU/L)	23.62 ± 14.31	23.46 ± 11.23	24.77 ± 10.98	26.63 ± 13.26	<.001
Iron (µmol/L)	12.28 ± 5.58	14.33 ± 5.70	14.87 ± 5.50	16.48 ± 5.88	<.001
LDH (IU/L)	151.93 ± 40.90	143.96 ± 33.54	137.65 ± 29.53	137.03 ± 39.51	<.001
Gender (%)					<.001
Male	147 (42.24%)	247 (42.51%)	217 (49.54%)	273 (53.63%)	
Female	201 (57.76%)	334 (57.49%)	221 (50.46%)	236 (46.37%)	
Race/ethnicity (%)					<.001
Non-hispanic white	183 (52.59%)	369 (63.51%)	291 (66.44%)	351 (68.96%)	
Non-hispanic black	80 (22.99%)	92 (15.83%)	45 (10.27%)	56 (11.00%)	
Mexican American	36 (10.34%)	38 (6.54%)	30 (6.85%)	30 (5.89%)	
Other race	49 (14.08%)	82 (14.11%)	72 (16.44%)	72 (14.15%)	
Arthritis (%)					<.001
Yes	199 (57.18%)	337 (58.00%)	215 (49.09%)	236 (46.37%)	
No	149 (42.82%)	240 (41.31%)	222 (50.68%)	271 (53.24%)	
Don’t know	0 (0.00%)	4 (0.69%)	1 (0.23%)	2 (0.39%)	
Gout (%)					.287
Yes	41 (11.78%)	60 (10.33%)	32 (7.31%)	47 (9.23%)	
No	306 (87.93%)	520 (89.50%)	406 (92.69%)	462 (90.77%)	
Don’t know	1 (0.29%)	1 (0.17%)	0 (0.00%)	0 (0.00%)	
Liver disease (%)					.038
Yes	34 (9.77%)	44 (7.57%)	23 (5.25%)	29 (5.70%)	
No	312 (89.66%)	537 (92.43%)	415 (94.75%)	479 (94.11%)	
Don’t	2 (0.57%)	0 (0.00%)	0 (0.00%)	1 (0.20%)	

Mean + SD/ N (%), for continuous variables: the *P* value was calculated by the weighted linear regression model. (%) for categorical variables: the *P* value was calculated by the weighted chi-square test.

ALP = alkaline phosphatase, ALT = alanine aminotransferase, AST = aspartate aminotransferase, BMI = body mass index, CPK = creatine phosphokinase, GGT = gamma glutamyl transferase, LDH = lactate dehydrogenase.

The relationship between ALB and bone ALP levels determined through multivariate regression analysis is shown in Table 2. Model 1, not adjusted; Model 2, adjusted for age, sex, race/ethnicity; Model 3, adjusted for the covariates presented in Table 1. In Model 1, ALB was negatively correlated to ALP (β = −2.147, 95%CI: [−2.542, −1.751], *P* < .001). After adjusting for confounders, the negative association was still present in Model 2 (β = −2.043, 95%CI: [−2.448, −1.638], *P* < .001) and Model 3 (β = −1.619, 95%CI: [−2.072, −1.166], *P* < .001). ALB levels were inversely correlated with bone ALP levels in all 3 models.

**Table 2:** The association between albumin (g/dL) and bone alkaline phosphatase (IU/L).

Exposure	Model 1 β (95% CI) *P* value	Model 2 β (95% CI) *P* value	Model 3 β (95% CI) *P* value
Albumin (g/dL)	−2.147 (−2.542, −1.751) < .001	−2.043 (−2.448, −1.638) < .001	−1.619 (−2.072, −1.166) < .001
Gender			
Male	−2.421 (−3.070, −1.772) < .001	−2.485 (−3.151, −1.818) < .001	−1.580 (−2.342, −0.818) .001
Female	−1.854 (−2.325, −1.383) < .001	−1.653 (−2.131, −1.176) < .001	−1.498 (−2.025, −0.971) < .001
Race/ethnicity			
Non-hispanic white	−1.984 (−2.496, −1.472) < .001	−1.948 (−2.472, −1.424) < .001	−1.603 (−2.193, −1.013) < .001
Non-hispanic black	−3.069 (−4.818, −1.320) .001	−3.367 (−5.157, −1.577) .033	−1.755 (−3.848, 0.338) .103
Mexican American	−1.910 (−2.807, −1.013) .004	−1.755 (−2.656, −0.854) .017	−1.092 (−2.136, −0.047) .041
Other race	−2.209 (−3.218, −1.200) .002	−2.192 (−3.212, −1.172) .003	−1.931 (−3.116, −0.746) .002
Liver disease			
Yes	−2.107 (−3.468, −0.746) .003	−2.475 (−3.920, −1.030) .011	−2.314 (−3.959, −0.668) .007
No	−1.928 (−2.336, −1.519) < .001	−1.794 (−2.212, −1.376) < .001	−1.519 (−1.991, −1.047) < .001
Arthritis			
Yes	−2.001 (−2.464, −1.537) < .001	−1.878 (−2.348, −1.409) < .001	−1.582 (−2.100, −1.064) < .001
No	−2.298 (−2.965, −1.631) < .001	−2.201 (−2.890, −1.512) < .001	−1.646 (−2.421, −0.871) .003
Gout			
Yes	−2.683 (−3.963, −1.404) .006	−2.617 (−3.911, −1.323) .0011	−0.676 (−2.061, 0.709) .341
No	−2.045 (−2.462, −1.629) < .001	−1.924 (−2.352, −1.497) < .001	−1.613 (−2.095, −1.130) < .001

Model 1: no covariates were adjusted. Model 2: age, sex, and race/ethnicity were adjusted. Model 3: age, sex, race/ethnicity, alanine aminotransferase, blood urea nitrogen, creatine phosphokinase, cholesterol, creatinine, gamma glutamyl transferase, glucose, phosphorus, total protein, uric acid, globulin, triglycerides, body mass index, aspartate aminotransferase. Iron lactate dehydrogenase, arthritis, gout, liver disease were adjusted. In the subgroup analysis stratified by sex, race/ethnicity, liver disease, arthritis and gout, the model is not adjusted for the stratification variable itself.

**Figure 1.**
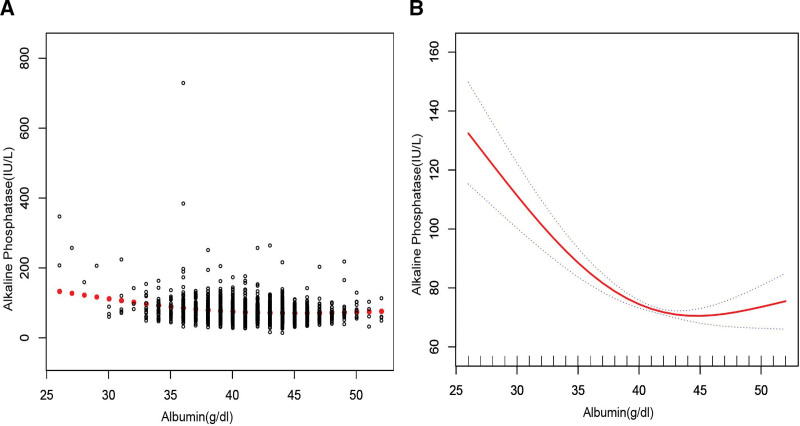
The association between serum albumin and alkaline phosphatase. (A) Each black point represents a sample. (B) Solid red line represents the smooth curve fit between variables. Gray dotted line represent the 95% of confidence interval from the fit. Age, sex, race/ethnicity, alanine aminotransferase, blood urea nitrogen, creatine phosphokinase, cholesterol, creatinine, gamma glutamyl transferase, glucose, phosphorus, total protein, uric acid, globulin, triglycerides, body mass index, aspartate aminotransferase, iron, lactate dehydrogenase, arthritis, gout, liver disease were adjusted.

**Figure 2.**
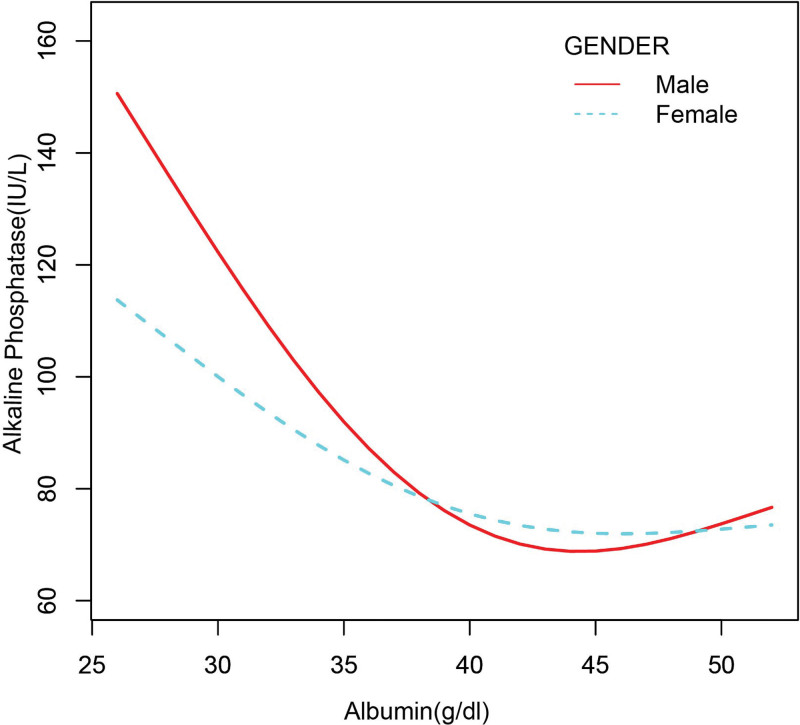
The association between serum albumin and alkaline phosphatase stratified by sex. Age, race/ethnicity, alanine aminotransferase, blood urea nitrogen, creatine phosphokinase, cholesterol, creatinine, gamma glutamyl transferase, glucose, phosphorus, total protein, uric acid, globulin, triglycerides, body mass index, aspartate aminotransferase. iron, lactate dehydrogenase, arthritis, gout, liver disease were adjusted.

**Figure 3.**
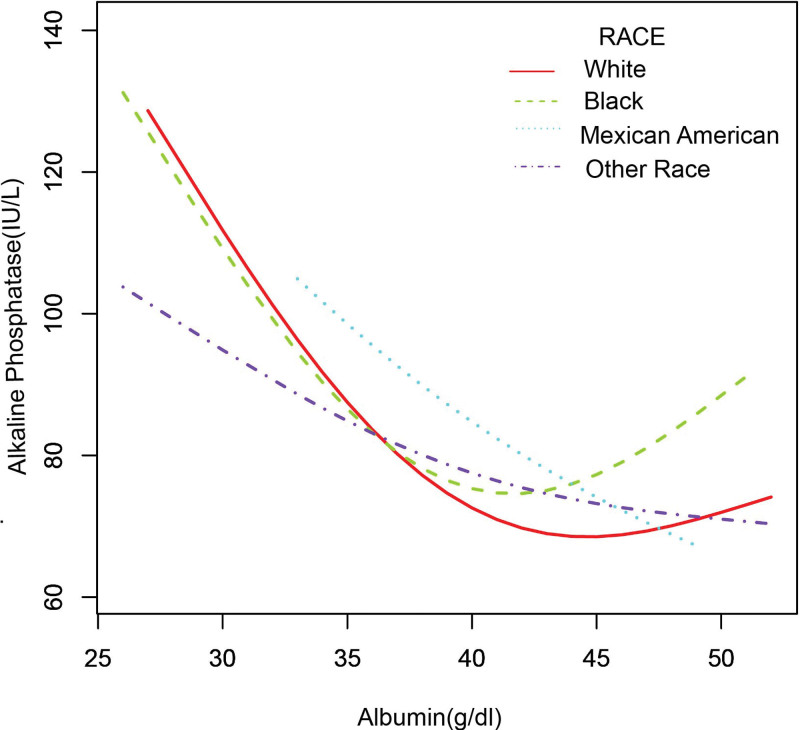
The association between serum albumin and alkaline phosphatase stratified by race/ethnicity. Age, sex, alanine aminotransferase, blood urea nitrogen, creatine phosphokinase, cholesterol, creatinine, gamma glutamyl transferase, glucose, phosphorus, total protein, uric acid, globulin, triglycerides, body mass index, aspartate aminotransferase. Iron, lactate dehydrogenase, arthritis, gout, liver disease were adjusted.

**Figure 4.**
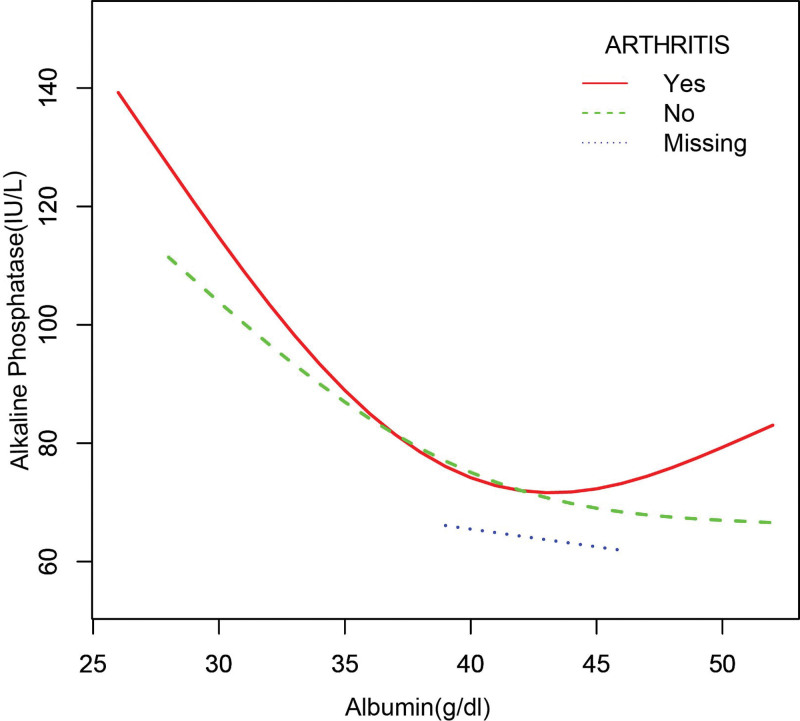
The association between serum albumin and alkaline phosphatase stratified by arthritis. Age, sex, race/ethnicity, alanine aminotransferase, blood urea nitrogen, creatine phosphokinase, cholesterol, creatinine, gamma glutamyl transferase, glucose, phosphorus, total protein, uric acid, globulin, triglycerides, body mass index, aspartate aminotransferase. Iron, lactate dehydrogenase, gout, liver disease were adjusted.

**Figure 5.**
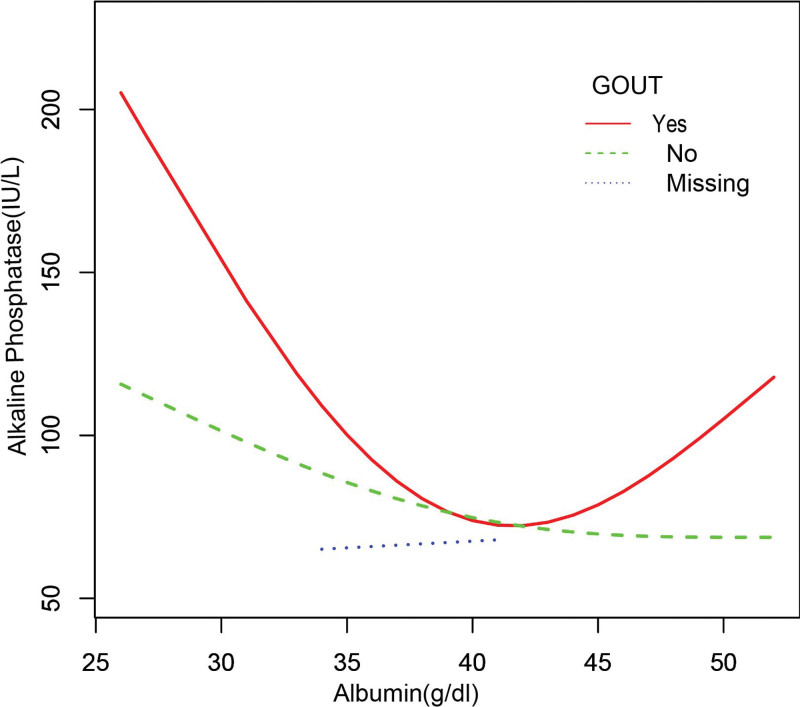
The association between serum albumin and alkaline phosphatase stratified by gout. Age, sex, race/ethnicity, alanine aminotransferase, blood urea nitrogen, creatine phosphokinase, cholesterol, creatinine, gamma glutamyl transferase, glucose, phosphorus, total protein, uric acid, globulin, triglycerides, body mass index, aspartate aminotransferase. Iron, lactate dehydrogenase, arthritis, liver disease were adjusted.

**Figure 6.**
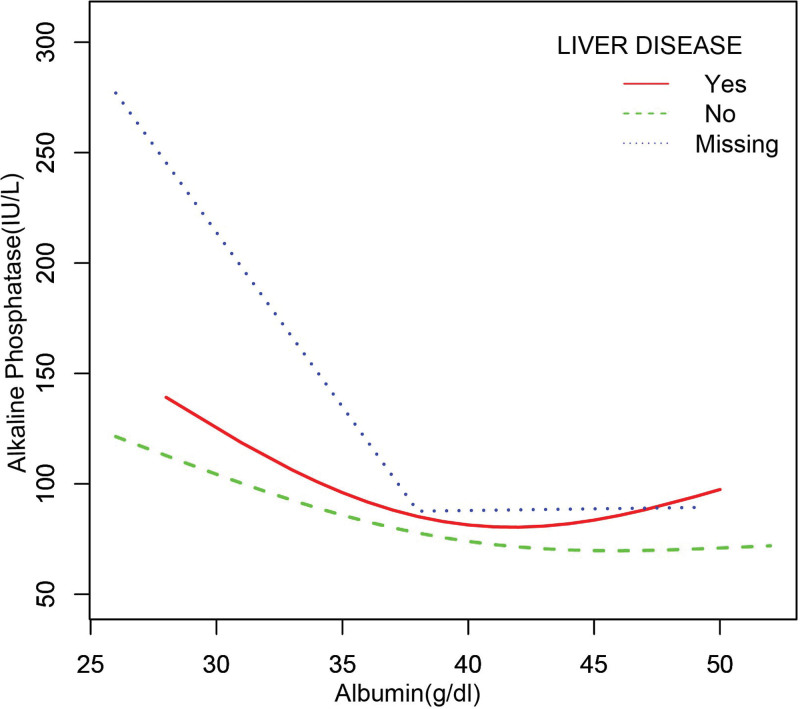
The association between serum albumin and alkaline phosphatase stratified by liver disease. Age, sex, race/ethnicity, alanine aminotransferase, blood urea nitrogen, creatine phosphokinase, cholesterol, creatinine, gamma glutamyl transferase, glucose, phosphorus, total protein, uric acid, globulin, triglycerides, body mass index, aspartate aminotransferase. Iron, lactate dehydrogenase, arthritis, gout, were adjusted.

In the subgroup analysis stratified by sex, race/ethnicity, liver disease, arthritis, and gout (Table 2), the negative correlation of ALB with bone ALP remained, except for black individuals (β = −1.755, 95%CI: [−3.848, 0.338], *P* = .103) and patients with gout (β = −0.676, 95%CI: [−2.061, 0.709], *P* = .340). The smooth curve fitting and generalized additive models used to describe the nonlinear relationship between ALB and bone ALP levels are shown in Figures 1–6. In black people and patients with gout, the relationship between ALB and ALP showed an inverted U-shaped curve, with an inflection point at approximately 42 g/dL.

## 4. Discussion

Our study examined the relationship between ALB and ALP levels using a representative sample of United States patients with cancer that were enrolled in NHANES 2011 to 2018. The results of this study showed that ALB was independently and negatively correlated with ALP, except in black patients and those with gout. In blacks and patients with gout, the correlation between ALB and ALP showed an inverted U-shaped curve with an inflection point of approximately 42 (g/dL).

ALB is an important nutritional indicator and is associated with the process of systemic inflammation.^[21]^ Additionally, ALB exerts antioxidant effects versus carcinogens.^[22]^ Therefore, the reduction in ALB will lead to a poor anticancer response and decreased immune function in patients with cancer, while also reflecting nutritional deficiency.^[23]^ In previous studies, ALB was shown to be a valuable prognostic marker and predictor of renal cancer, prostate cancer, hepatocellular carcinoma, and various other cancers.^[24,25]^ ALP is a hydrolase that is converted in the liver, kidney, and bile ducts and is commonly associated with bone metastases and liver disease.^[17]^ The use of ALP as a tumor marker dates to the 1980s.^[26]^ Since then, hyperphosphatemia (i.e., elevated ALP levels) has been proposed as a prognostic marker for various cancers, including prostate cancer,^[27]^ renal cell carcinoma,^[28]^ liver cancer,^[29]^ gastric cancer,^[30]^ and pancreatic cancer.^[31]^ In particular, the sensitivity, specificity and accuracy of ALP in predicting bone metastasis of cancer are more than 70%; therefore, it has important value in the prediction of tumor bone metastases and is an independent prognostic indicator in patients with bone metastasis.^[9,10]^ Among our representative United States population, a higher ALB level was associated with a lower ALP level in most patients with cancer. Considering this association, ALB may be a potential predictive biomarker in patients with bone metastases. Therefore, measurement of ALB levels can serve as a screening tool for bone metastases while guiding therapeutic interventions.

Currently, clinical studies on the association between ALB and ALP in patients with cancer are limited, and most studies have focused on the ALB/ALP ratio. Several studies have focused on the association between a low ALB/ALP ratio and poorer overall survival.^[16,17,32]^ To our knowledge, this is the first time that the association between ALB and ALP in patients with cancer has been assessed using different multivariate logistic regression models, and the robustness of the results has been confirmed by subgroup analysis stratified by sex, ethnicity, liver disease, arthritis, and gout. Our findings suggest that higher ALB levels are associated with lower ALP levels in patients with cancer, providing a basis for the prediction and treatment of bone metastases in patients with cancer. At the same time, our findings may help clinicians distinguish high-risk cancer patients before implementing treatment. Patients with low ALB may have elevated ALP, reduced immunity, malnutrition and increased treatment resistance. Prompt intervention in patients with low ALB can improve treatment outcomes. Meanwhile, patients with low ALB may require more additional radiotherapy or chemotherapy, as an increase in ALP may reflect bone metastases or micrometastasis. In addition, for the first time, our subgroup analysis stratified by race and gout revealed an inverted U-shaped association between ALB and ALP levels in black patients with gout. Large prospective studies are needed to clarify the relationship between ALB and ALP levels in black patients and those with gout.

However, the limitations of our study should be acknowledged. First, the number of included studies and the sample size were limited; fluctuations over time can cause changes in the optimal cutoffs of the markers. Especially when it comes to individual samples, the best cutoff may be different. Therefore, the results of this study need to be confirmed by further prospective studies with significantly larger sample sizes. Second, owing to the cross-sectional design of this study, determining whether a causal relationship exists between ALB and ALP levels is difficult. Third, confounding factors not included in this study may have influenced the results.

## 5. Conclusion

Our study showed an inverse correlation between ALB and ALP levels in most patients with tumors, but not in black patients and those with gout. In those groups, the association between ALB and ALP showed an inverted U-shaped curve, with an inflection point of approximately 42 g/dL. Overall, our findings indicate that the measurement of ALB levels can serve as a screening tool for bone metastases while guiding therapeutic intervention.

## Author contributions

**Conceptualization:** Yiqian Jiang, Zhaoyang Li.

**Data curation:** Yiqian Jiang, Zhaoyang Li.

**Methodology:** Zhaoyang Li.

**Software:** Yong Cai, Yingying Ding.

**Writing – original draft:** Yong Cai, Yingying Ding, Xiangyang Kong.

**Writing – review & editing:** Zhaoyang Li.
